# Supplementary material for: Exploring synchronous, asynchronous, and conventional online courses in higher education
Source: PLoS One. 2026 Mar 31;21(3):e0345955. doi: 10.1371/journal.pone.0345955 (PMC13038016; doi:10.1371/journal.pone.0345955)
Supplement: S1 File — Detailed description of the variables and data coding. (DOCX) [file pone.0345955.s002.docx]

**Data Description for “Exploring Synchronous, asynchronous, and conventional online courses in higher education”**

**1. Overview**

This dataset contains the anonymized survey responses used in the study examining learners’ perceptions of synchronous (Syn), asynchronous (Asyn), and conventional online courses (COC) based on an extended Information Systems Success Model (ISSM). The dataset includes all variables necessary to reproduce the analyses reported in the manuscript.

**2. File Contents**

- Data file: online_learning_dataset (n=795).xlsx

- Sheet name: data

- Number of cases: 795

- Number of variables: 50

**3. Variable Description**

| **Variable name** | **Description** | **Scale** | **Notes** |
| --- | --- | --- | --- |
| ID | Case Identifier | Nominal | Randomized ID |
| Group | Online Learning Modality | Nominal | 1 = Syn, 2 = Asyn, 3 = COC |
| SQ1–SQ4 | System Quality items | Likert 1–5 | 1 (Strongly Disagree) – 5 (Strongly Agree) |
| IQ1-IQ6 | information quality | Likert 1–5 | 1 (Strongly Disagree) – 5 (Strongly Agree) |
| SE1-SE8 | academic self-efficacy | Likert 1–5 | 1 (Strongly Disagree) – 5 (Strongly Agree) |
| TP1-TP15 | teaching presence | Likert 1–5 | 1 (Strongly Disagree) – 5 (Strongly Agree) |
| PU1-PU3 | perceived usefulness | Likert 1–5 | 1 (Strongly Disagree) – 5 (Strongly Agree) |
| LS1-LS8 | learning satisfaction | Likert 1–5 | 1 (Strongly Disagree) – 5 (Strongly Agree) |
| CI1-CI4 | continuance intention to use | Likert 1–5 | 1 (Strongly Disagree) – 5 (Strongly Agree) |

**4. Data Collection Procedure**

- Data were collected from undergraduate students enrolled in higher education courses in South Korea.

- Participants completed an online survey administered through the institutional Learning Management System (LMS).

- Respondents were instructed to answer based solely on the specific course through which they accessed the survey link.

- All responses are anonymous; no personally identifiable information was collected.

**5. Data Processing**

- No transformations, imputations, or recoding were applied beyond removing incomplete cases.

- Likert-type items were retained in their original scale (1–5).

- Modality coding is Syn=1, Asyn=2, COC=3.

**6. Ethical Considerations**

This study was exempt from Institutional Review Board (IRB) review under the Bioethics and Safety Act of the Republic of Korea. All participants provided informed consent.

**7. Contact Information**

Won Sug Shin

Incheon National University

Email: wsshin@inu.ac.kr
