# Supplementary material for: Exploring synchronous, asynchronous, and conventional online courses in higher education
Source: PLoS One. 2026 Mar 31;21(3):e0345955. doi: 10.1371/journal.pone.0345955 (PMC13038016; doi:10.1371/journal.pone.0345955)
Supplement: Attachment — Submitted filename: response_to_reviewer _1215.docx [file pone.0345955.s004.docx]

R**esponse to Reviewer**

**Reviewer #1:**

1. The title should be re-phrased to something like “Exploring the Synchronous, asynchronous, and conventional online courses in higher education”:

: Thank you for your comment and offering an attractive title of the manuscript. The title has been revised accordingly to:

“Exploring synchronous, asynchronous, and conventional online courses in higher education.”

2. The study research design, research questions/objectives and methodology are not stated in the abstract:

: We agree with this comment. The abstract has been substantially revised to explicitly state the research design, key research questions, analytical methods (latent mean analysis and multi-group analysis), and sample characteristics.

3. Five or more keywords should be added as there is no keywords list after the abstract:

: A keywords list has now been added after the abstract. The keywords included are:

“**Keywords**: online learning, synchronous learning, asynchronous learning, conventional online course, multi-group analysis”

Copy editing is recommended:
: Thank you for your comment. The manuscript has been thoroughly copyedited to enhance clarity and language accuracy and to ensure full compliance with the PLOS ONE formatting and style requirements.

4. In the Methodology section, within the Participants subsection, lines 226-7, the author stated that the whole number of respondents is 795, showing “313 students reported experience with Syn, 316 with Asyn, and 166 with 227 COC”. Aren’t there cases where some respondents experienced more than one mode? Is it really they experienced ONLY one mode of learning? The whole world experienced the three modes, didn’t they?:

: Thank you for your comment. We addressed this problem.

5. In line 279, there is a typing error:

: Thank you for catching this typo. In the revised manuscript, we fixed this typo in line 279(differences) and 281 (followed). In addition, we thoroughly read the manuscript to find other typos.

6. Titles of table captions are not to end with a full stop which is usually used to end a full sentence.

: Thank you for your comment. All table captions have been reviewed and revised to ensure that they do not end with a full stop, in accordance with PLOS ONE style guidelines.

7. It is highly suggested to have a section titled ’Limitations and suggestions for further work’ before the Conclusion section.

: We thank the reviewer for this helpful suggestion. In the revised manuscript, we have added a new dedicated section titled “Limitations and Suggestions for Further Work” immediately before the Conclusions section to explicitly discuss the study’s limitations and directions for future research.

8. The conclusion section is weak in the sense that it does nor truly conclude the present study. The author is invited to re-work on this bringing the main findings of this study.

: We appreciate this important comment. The conclusion section has been substantially rewritten. The revised conclusion has been substantially expanded to synthesize the main empirical findings, explicitly reflect modality-specific differences identified through latent mean analysis and multi-group SEM, and articulate clearer theoretical and practical implications based on the extended ISSM framework.

9. There is no reason why Figure 2 presented before figure 1 at the end of the paper.

: Thank you for noting this. The figures have been reordered so that Figure 1 is presented before Figure 2, and all figure citations have been checked for consistency.

**Reviewer #2:**

1. Introduction
   Since COVID-19 is no longer a pressing issue in online education, the introduction should focus on the broader development and sustainability of online learning rather than positioning the pandemic as the central rationale.

: We appreciate the reviewer’s insightful comment and agree that online learning should be framed within a broader, long-term perspective. In the revision, following your suggestion, we re-organized the introduction section to more clearly emphasize the need for continued research to support the advancement and sustainability of online learning.

2. Literature Review
   Some of the cited references are relatively outdated (e.g., Skylar, 2009; Sun & Wu, 2016), and it is recommended that the authors include more recent studies.
   The discussion of synchronous, asynchronous, and conventional online courses remains largely descriptive, without synthesizing the literature to highlight a clear research gap.
   The review does not explicitly state the research questions or explain how this study addresses the theoretical or methodological limitations identified in previous work.

: Thanks for the constructive and insightful comments regarding the Literature Review section.

First, in response to the concern about outdated references, we have carefully reviewed and updated the literature by incorporating a range of more recent and relevant studies, while retaining earlier foundational works where theoretically appropriate.

Second, we have substantially revised the Literature Review to move beyond a descriptive overview toward a more integrative and analytical synthesis. The revised sections now explicitly compare prior findings across the three modalities, identify areas of inconsistency and limitation in existing studies.

Third, based on this synthesis, we have explicitly articulated the theoretical and methodological gaps addressed by the present study. In particular, we emphasize the limited application of the Information Systems Success Model (ISSM) to comparative analyses across online learning modalities, as well as the scarcity of studies examining learners’ satisfaction and continuance intention using both latent mean analysis and multi-group structural equation modeling. The research questions are now clearly stated and directly linked to these identified gaps at the end of the Introduction.

Overall, we believe these revisions strengthen the coherence of the Literature Review and clarify how the present study advances prior research by providing a theoretically grounded and methodologically rigorous comparison of synchronous, asynchronous, and conventional online courses.

3. Data Analysis
   The logical connection between latent mean analysis (LMA) and multi-group SEM remains unclear. Although the analytical sequence is correct, the authors should specify which group served as the reference in LMA and elaborate on how the three analytical steps are theoretically linked.
   While the authors report CFI, TLI, and RMSEA values and mention ΔCFI/ΔTLI/ΔRMSEA thresholds, the actual Δ values and references for these cut-offs are not provided. Including a summary table and relevant citations (Cheung & Rensvold, 2002; Chen, 2007) would enhance transparency.
   Finally, please clarify whether the reported fit indices meet accepted benchmarks (CFI/TLI > 0.90, RMSEA < 0.08) and consider reporting standard errors or confidence intervals for path coefficients to strengthen interpretability.

: Thanks for these important comments.

First, we have clarified the logical and theoretical linkage between latent mean analysis (LMA) and multi-group structural equation modeling (SEM) in the revised manuscript. Specifically, we now explicitly state that the conventional online course (COC) group served as the reference group in the latent mean analysis. We also elaborate on how LMA was used to identify modality-level differences in perceived constructs, which was subsequently followed by multi-group SEM to examine whether the underlying structural relationships among ISSM variables operated equivalently across modalities.

Second, to enhance analytic transparency, we now report the actual ΔCFI, ΔTLI, and ΔRMSEA values used in the invariance testing procedure and include them in a summary table. In addition, we have added explicit references to established cut-off criteria for model comparison, including Cheung and Rensvold (2002) and Chen (2007).

Third, we have clarified that all reported fit indices meet commonly accepted benchmarks (CFI/TLI > 0.90, RMSEA < 0.08), and this information is now explicitly stated in the revised Results section.

We believe that these revisions substantially strengthen the clarity, rigor, and transparency of the analytical procedures employed in this study.

4. Discussion and Conclusion

The discussion section interprets the findings largely from an empirical perspective but does not fully draw on the Information System Success Model (ISSM) and its extended variables to explain the underlying mechanisms. The authors are encouraged to explicitly link the observed relationships (e.g., stronger PU→CI and IQ→LS paths in the COC mode) to the ISSM framework to enhance theoretical coherence.

Some explanations (e.g., “teaching presence increases satisfaction in the Syn mode” or “COC performs best overall”) extend beyond what is directly supported by the statistical evidence.

Although the conclusion summarizes the main implications, the discussion remains rather general. The authors could make the implications more specific and actionable, for example, by suggesting how improved information integration and system usability could strengthen the PU→CI relationship observed in the COC mode.

: Thank you for this helpful comment. To address this comment, we have tried to expand our discussion in several ways.

The manuscript has been revised as follows:

- **Stronger theoretical integration with ISSM**
  - The Discussion section has been substantially revised to explicitly interpret the findings within the extended Information Systems Success Model (ISSM).
  - Key structural relationships (e.g., PU→CI, IQ→LS, PU→LS, IQ→PU) are now directly linked to the ISSM’s sequential mechanism (quality perceptions → perceived usefulness → satisfaction → continuance intention).
  - Modality-specific differences are discussed as variations in how this ISSM mechanism operates across Syn, Asyn, and COC environments, enhancing theoretical coherence.
- **Alignment of interpretations with statistical evidence**
  - All explanations were carefully reviewed to ensure they are fully supported by the empirical results.
  - Statements implying causal effects or general superiority (e.g., “COC performs best overall” or unconditional claims about teaching presence) were removed or reframed.
  - The revised ‘Discussion’ now interprets findings based on latent mean differences and structural path coefficients, supported by existing literature, without overstating effects.
- **More concrete and actionable implications**
  - The Discussion and Conclusion sections now provide specific, practice-oriented implications rather than general recommendations.
  - In particular, the strong PU→CI relationship in the COC modality is discussed in terms of improving the clarity and structural organization of instructional information and enhancing system usability.
  - Additional actionable suggestions include incorporating targeted synchronous elements into Asyn and COC designs and providing structured instructional design templates to support sustainable online learning.

We believe these revisions strengthen both the theoretical grounding and the practical relevance of the Discussion and Conclusion sections.
