# Supplementary material for: Exploring synchronous, asynchronous, and conventional online courses in higher education
Source: PLoS One. 2026 Mar 31;21(3):e0345955. doi: 10.1371/journal.pone.0345955 (PMC13038016; doi:10.1371/journal.pone.0345955)
Supplement: Attachment — Submitted filename: Response to Reviewers.docx [file pone.0345955.s005.docx]

R**esponse to Reviewer**

**Reviewer #2:**

1. While the research questions are now clearly stated, the authors may consider explicitly mapping each research question to the corresponding analytical results or subsections in the Results or Discussion sections. Making this linkage more explicit would further improve readability and help readers more easily follow how each research question is addressed empirically.

Response : We appreciate this helpful suggestion. To make the analytical structure more explicit, we revised the manuscript in two complementary ways. First, at the beginning of the Results section, we added brief signposting sentences that clearly indicate how each research question was addressed empirically (RQ1 through latent mean analysis and RQ2 through multi-group structural equation modeling). Second, at the beginning of the Discussion section, we clarified that the discussion is explicitly organized around these two research questions, linking RQ1 to the interpretation of latent mean differences and RQ2 to the examination of modality-specific structural relationships. We believe these revisions improve readability and help readers more easily follow how each research question is addressed across sections.

2. The revised Discussion appropriately focuses on the significant structural relationships. However, a brief reflection on non-significant or weaker paths could further strengthen the interpretation.

Response : We appreciate this insightful comment. In response, we added a brief integrative paragraph in the Discussion that reflects on non-significant or relatively weaker structural paths observed across learning modalities. Rather than reiterating the detailed interpretations of individual paths already discussed earlier, this paragraph provides a higher-level, modality-sensitive perspective that situates these weaker or non-significant relationships within broader instructional and contextual characteristics of synchronous, asynchronous, and conventional online courses. This addition is intended to enhance the interpretive completeness of the Discussion while remaining cautious and fully grounded in the reported results.
